# Supplementary figures and images for: The short-term effect of residential home energy retrofits on indoor air quality and microbial exposure: A case-control study
Source: PLoS One. 2021 Sep 20;16(9):e0230700. doi: 10.1371/journal.pone.0230700 (PMC8452058; doi:10.1371/journal.pone.0230700)

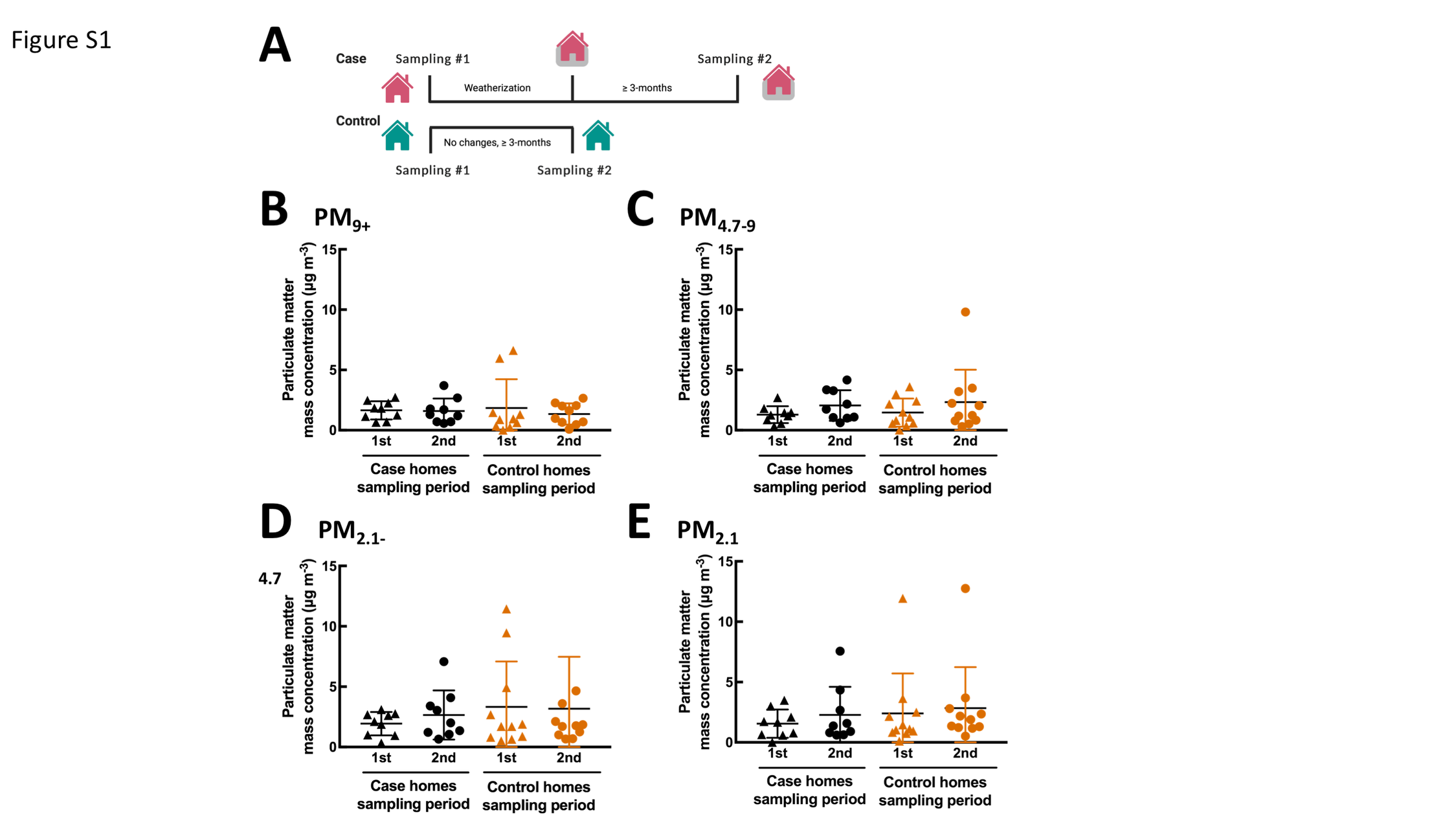

Supplement: S1 Fig — Sampling strategy (A) for case and control homes. Mass concentration of particulate matter size bins (B: PM9+; C: PM4.7–9; D: PM2.1–4.7; E: PM2.1) across all homes and sampling periods. Points are colored based on house type, with case homes as black points and control homes as orange points. Triangles represent the first sampling period, and circles represent the second sampling period. Significance test via the paired t-test indicated no significant difference between sampling periods in both case and control homes. (TIF) [file pone.0230700.s001.tif]

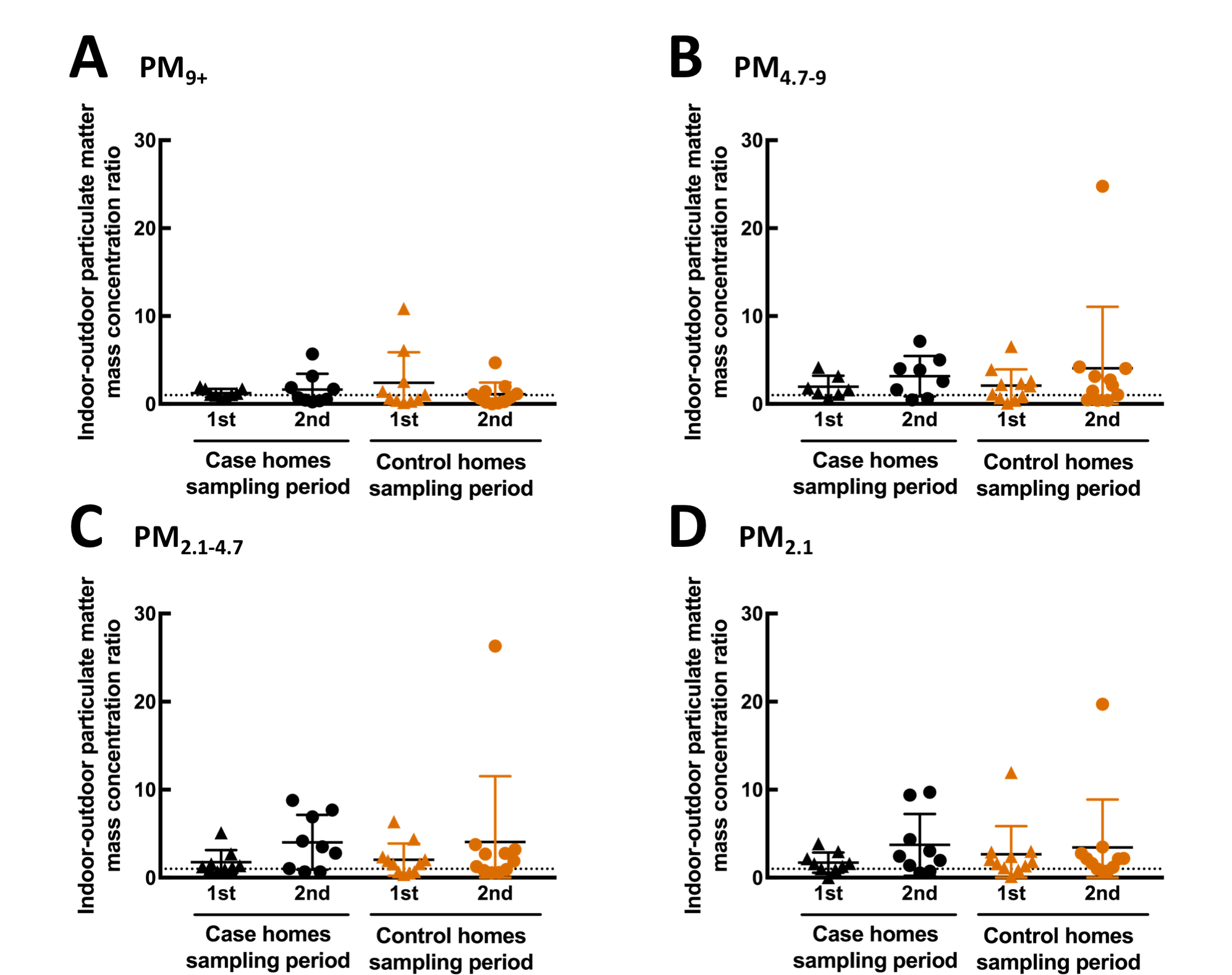

Supplement: S2 Fig — Indoor-outdoor concentration ratio of particulate matter size bins (A: PM9+; B: PM4.7–9; C: PM2.1–4.7; D: PM2.1) across all homes and sampling periods. IO ratio is defined as the indoor particulate matter mass concentration over the outdoor mass concentration. Black points represent case homes and orange points represent control homes. Triangles represent the first sampling period, and circles represent the second sampling period. A dashed line represents a 1:1 ratio. Significance test via the paired t-test indicated no significant difference between sampling periods in both case and control homes. (TIF) [file pone.0230700.s002.tif]

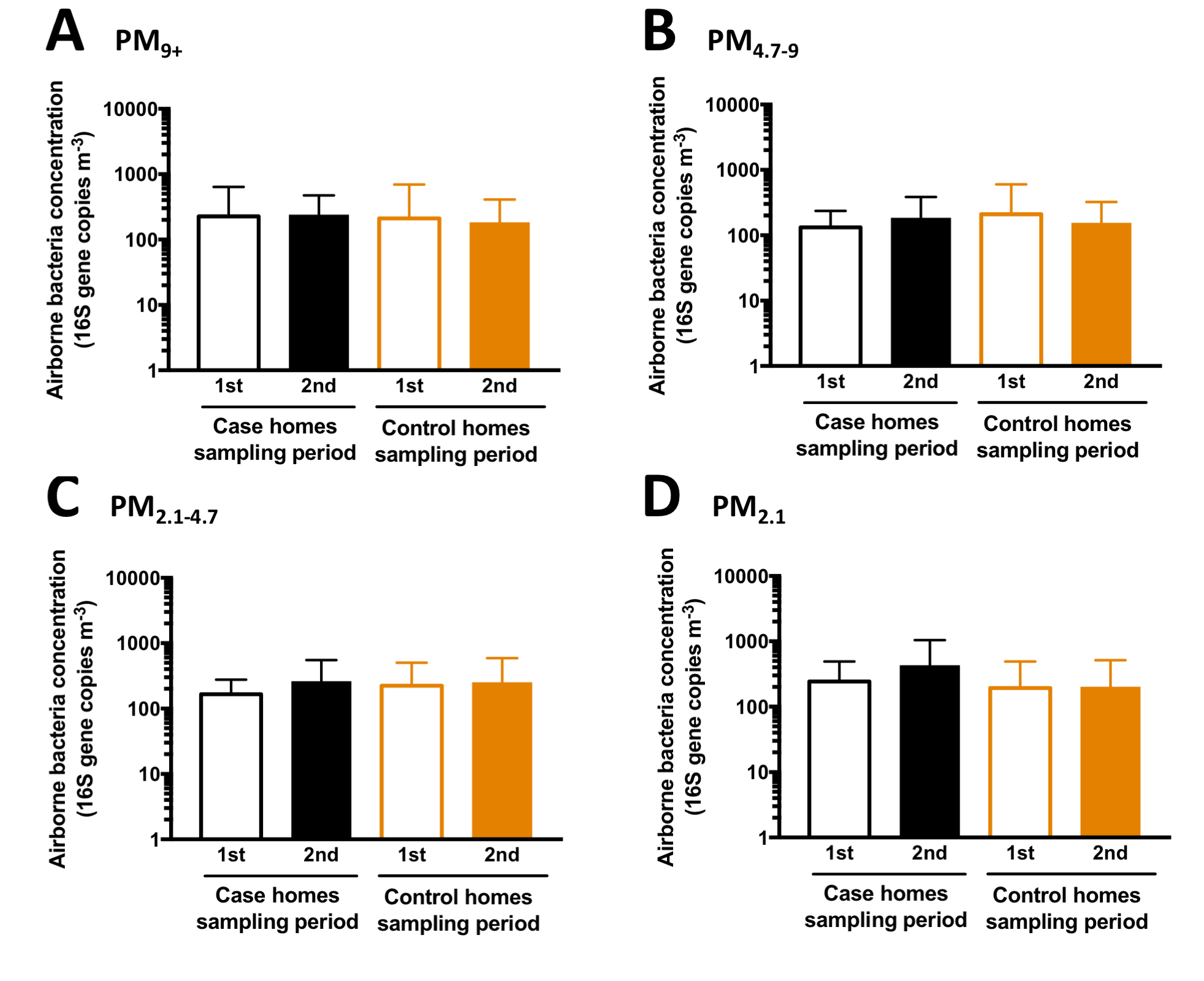

Supplement: S3 Fig — Airborne bacterial concentration in four particulate matter size bins (A: PM9+; B: PM4.7–9; C: PM2.1–4.7; D: PM2.1) across all homes and sampling periods. Black points represent case homes and orange points represent control homes. Empty bars represent the first sampling period, and filled bars represent the second sampling period. Significance test via the paired t-test indicated no significant difference between sampling periods in both case and control homes. (TIF) [file pone.0230700.s003.tif]

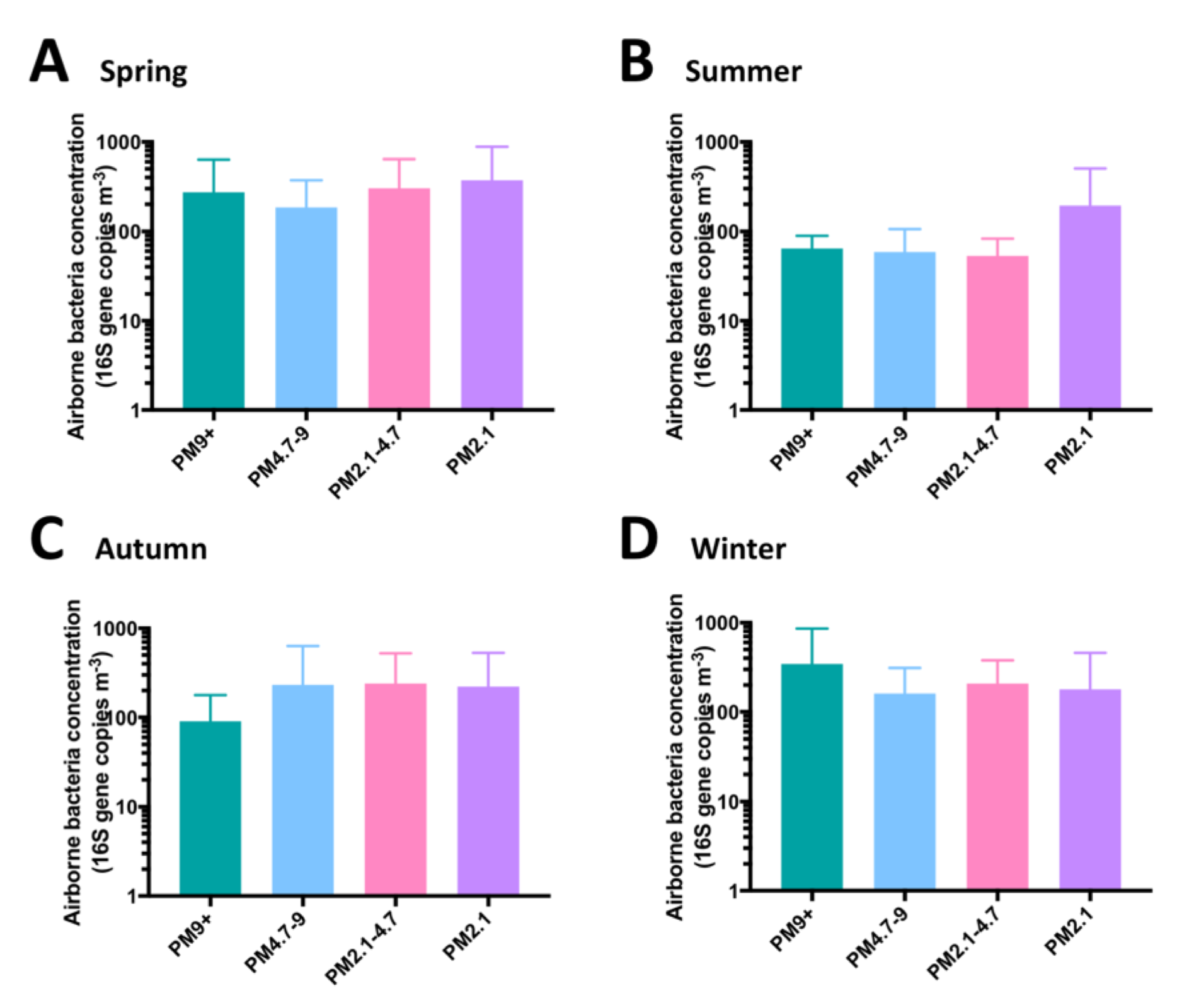

Supplement: S4 Fig — Concentration of bacteria in indoor air across all homes, in 16S gene copies per m3. All air samples from both sampling periods were consolidated and categorized by size bins for each season. Bars are colored based on size bin, with the following grouping: PM9+: green; PM4.7–9: blue; PM2.1–4.7: pink; PM2.1: purple. The unpaired t-test was used to determine significance between seasons. No significant were found between groups. (TIF) [file pone.0230700.s004.tif]

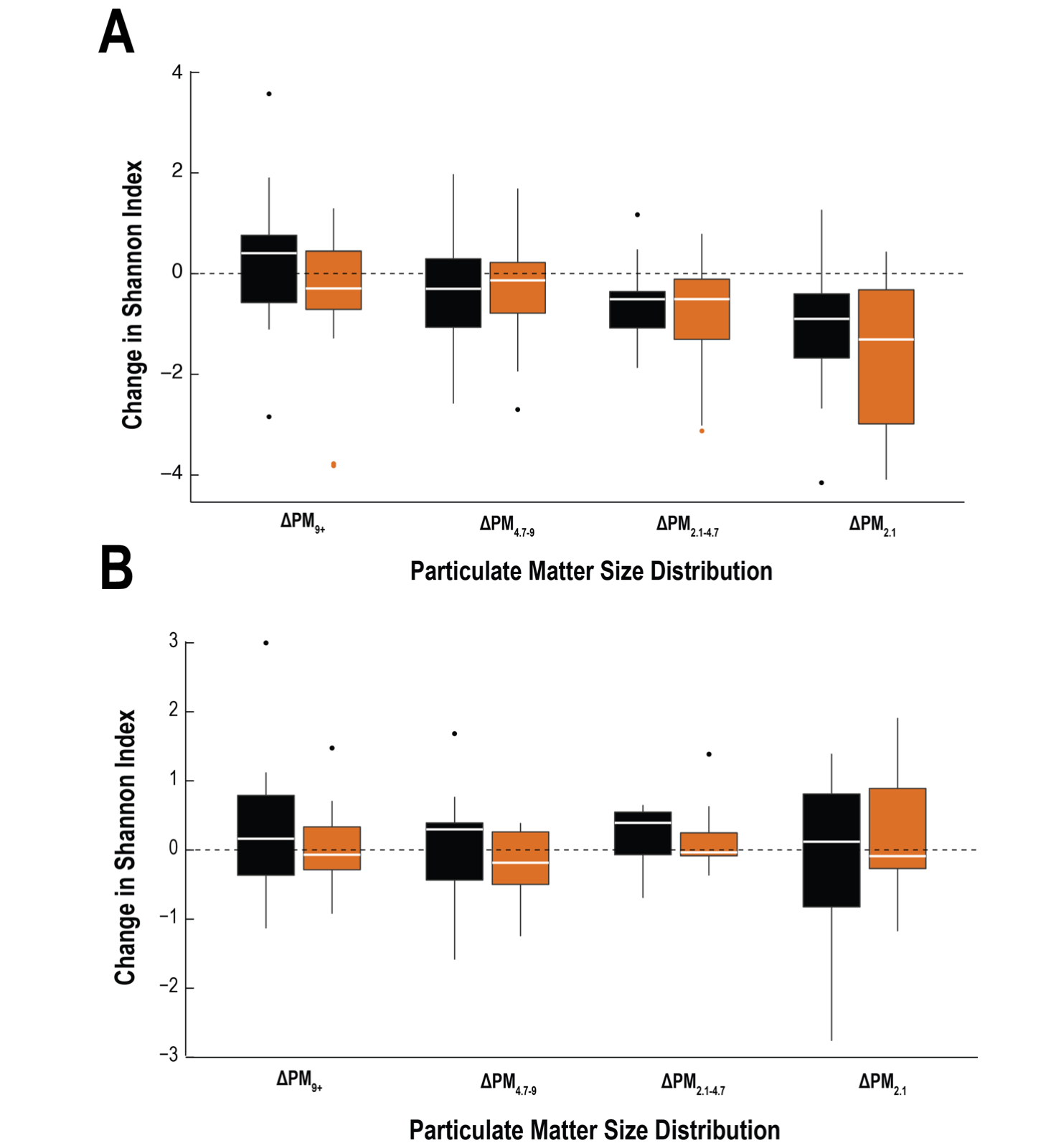

Supplement: S5 Fig — Community diversity is reported as a change in the Shannon index for airborne bacteria samples in both the case (black) and control (orange) homes. A) Outdoor-indoor alpha diversity comparison of airborne bacterial communities, separated into particle size bins. Change in Shannon Index was calculated as outdoor air minus indoor air alpha diversity for each home. Therefore, negative values indicate greater diversity indoors compared to outdoors. No significance was found between case and control homes. B) Differences in alpha diversity of indoor air bacterial community, calculated by subtracting the first sampling alpha diversity from the alpha diversity of the second sampling. There is no significance between case and control homes across all air particle size bins. (TIF) [file pone.0230700.s005.tif]

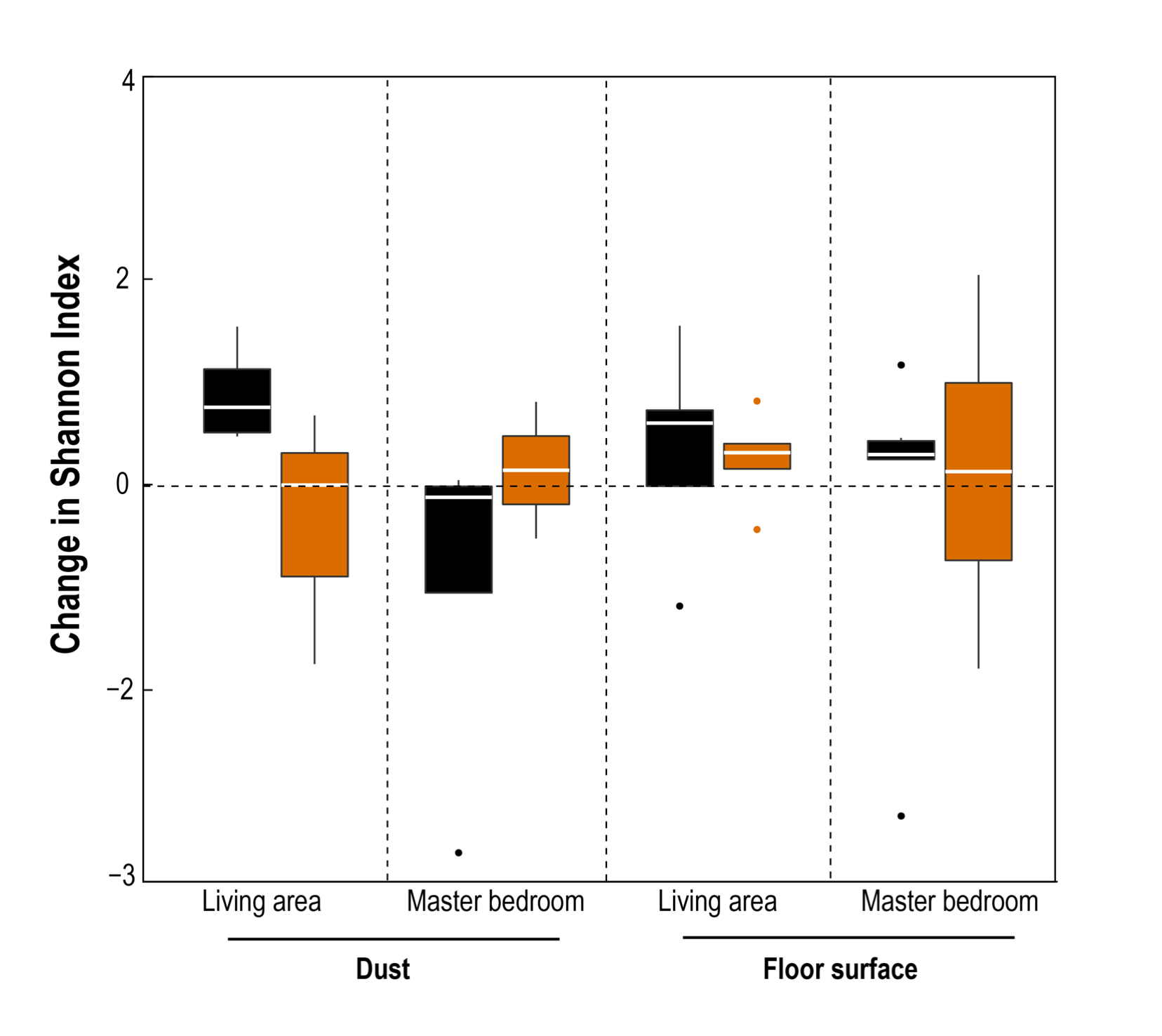

Supplement: S6 Fig — Bacterial community diversity is reported as a change in the Shannon Index for airborne bacterial samples in both the case (black) and control (orange) homes. Differences in alpha diversity of surface and dust bacterial communities, calculated by subtracting the first sampling alpha diversity from the alpha diversity of the second sampling. (TIF) [file pone.0230700.s006.tif]

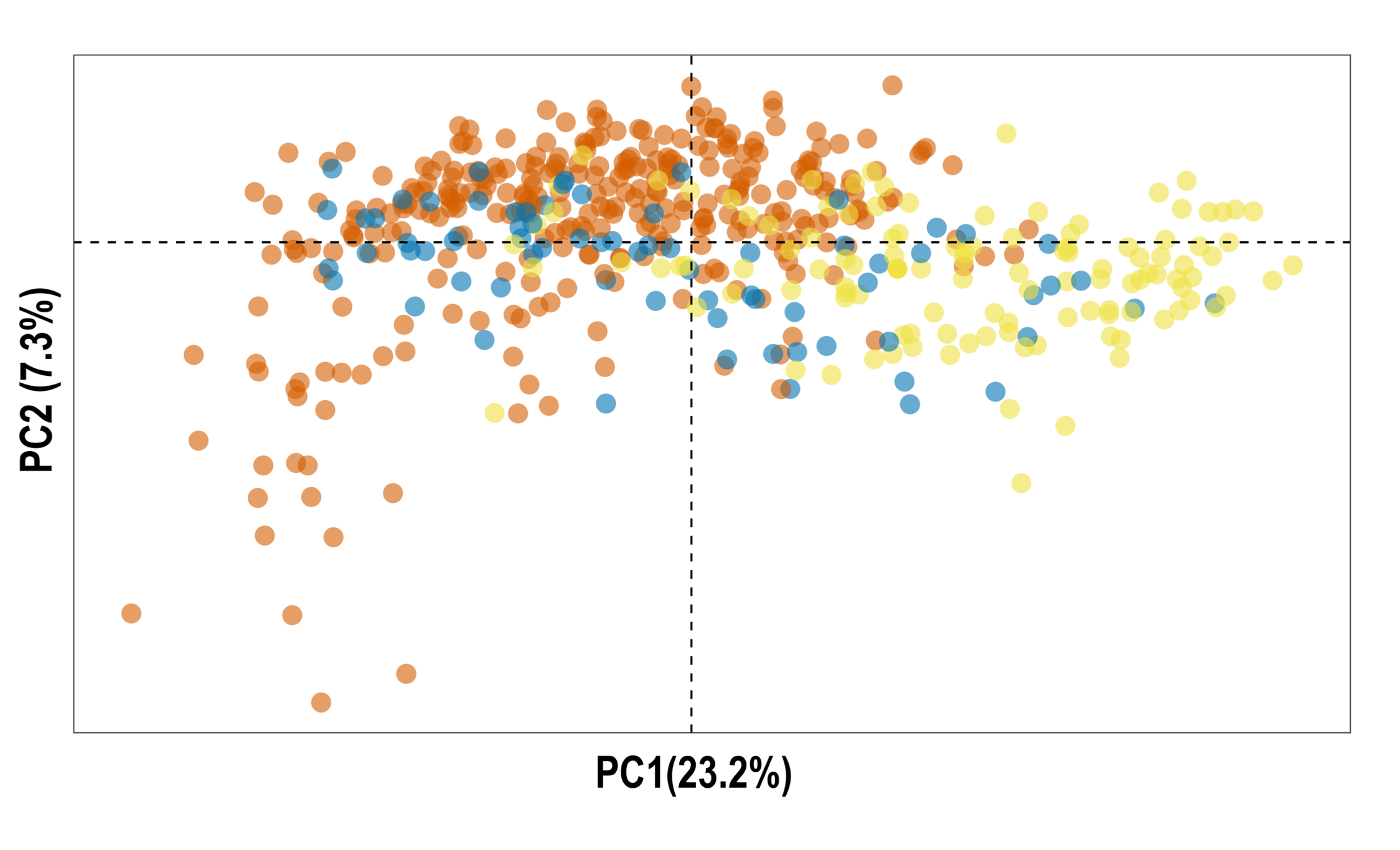

Supplement: S7 Fig — Principal coordinates analysis with the weighted UniFrac dissimilarity metric of all air, dust, and surface samples. Only the first two axes are shown. Dissimilarity distances are calculated from the differences in taxonomic composition and abundance between samples. Each point represents one sample, colored by sample source: air (turquoise), carpet dust (yellow), and floor surface (blue). (TIF) [file pone.0230700.s007.tif]

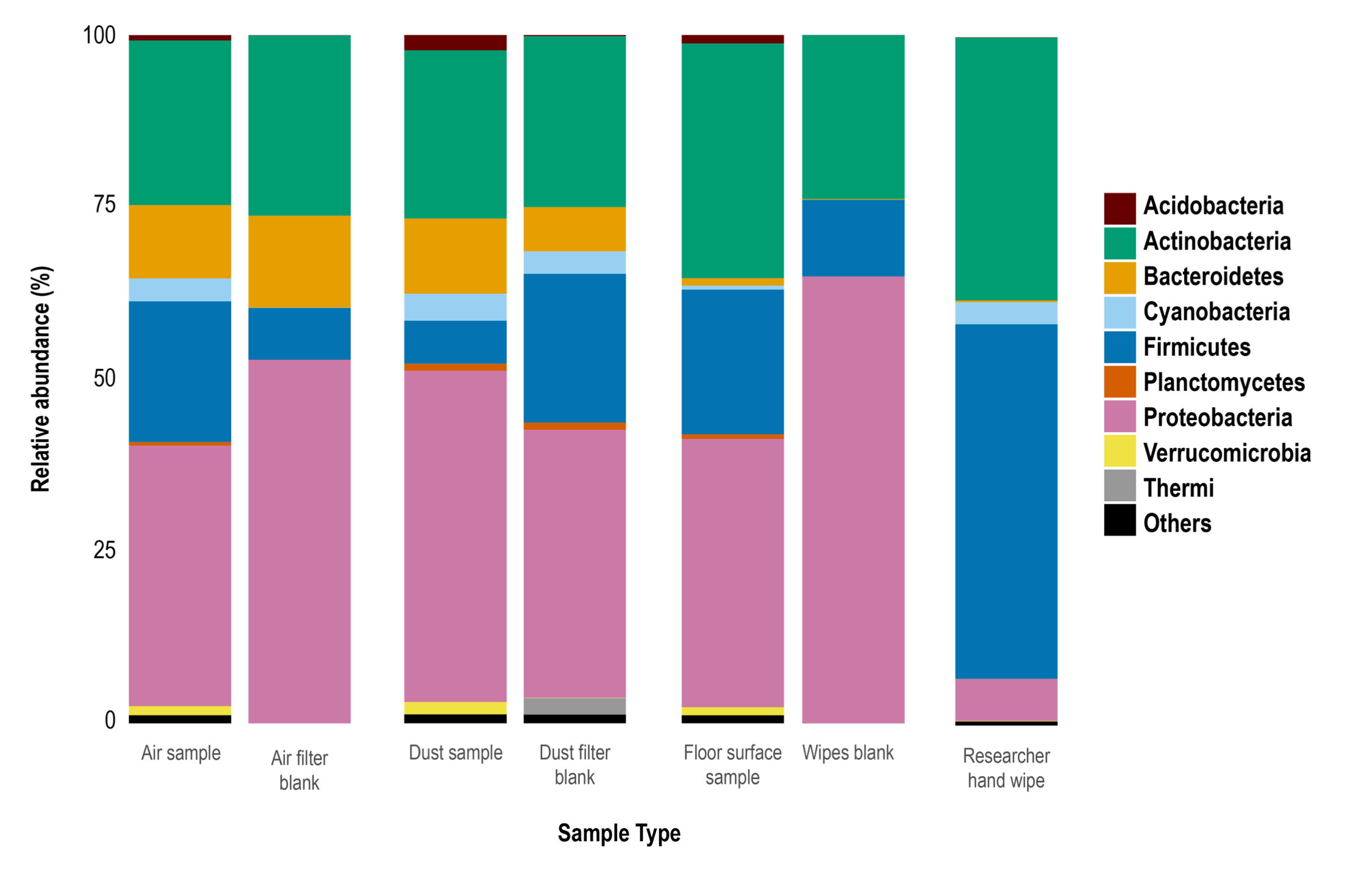

Supplement: S8 Fig — Indoor airborne bacterial communities in each air particles size bins were compared to their corresponding outdoor airborne bacterial communities (turquoise). The same indoor air samples were also compared to living room floor surface (yellow) and living room carpet dust (blue) communities. Positive values indicate that two communities are more dissimilar in the second sampling period compared to the first sampling period. Negative values indicate that the two communities became more similar. NS: no significant. (TIF) [file pone.0230700.s008.tif]

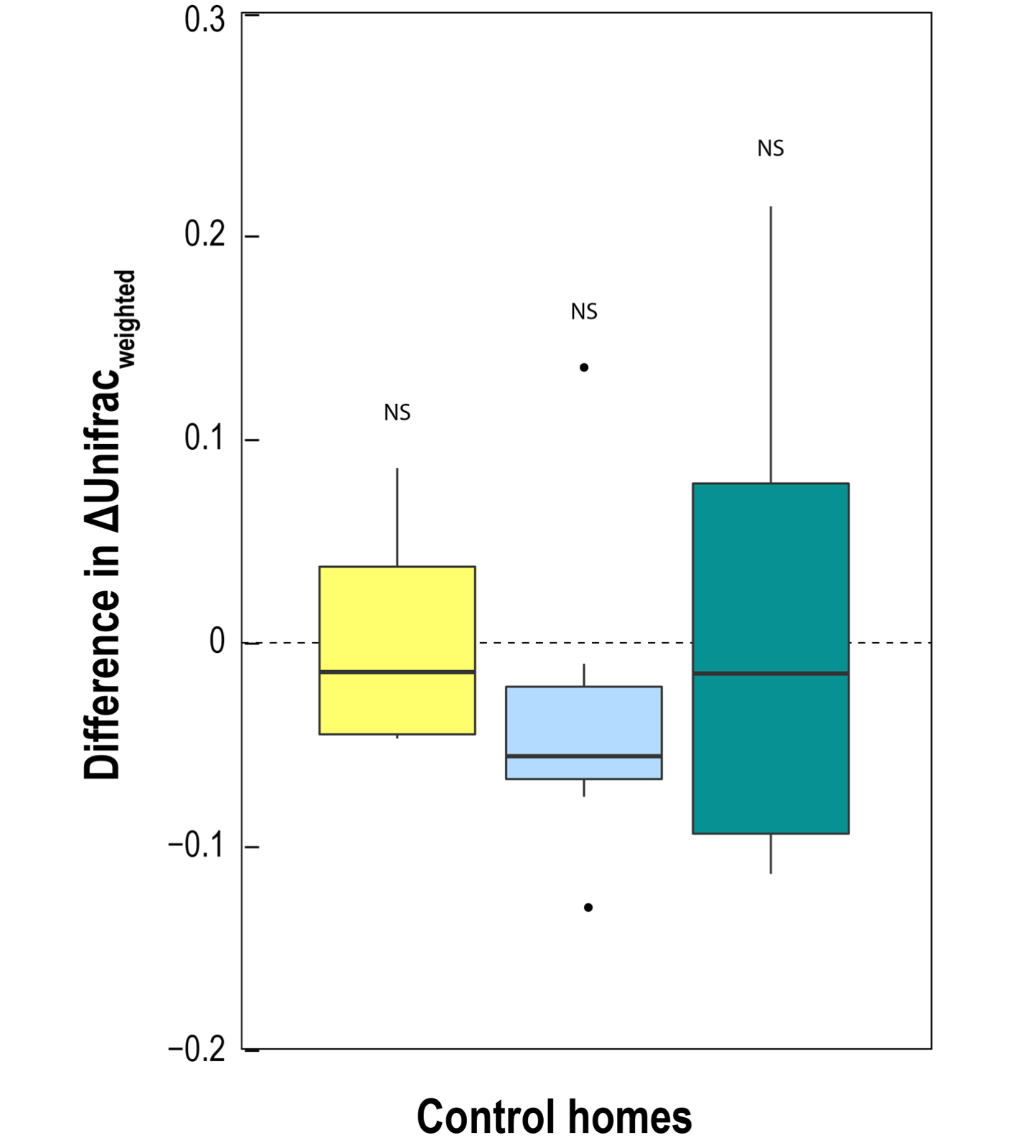

Supplement: S9 Fig — 16S rRNA gene sequences summarized at the phylum level of home samples (air, carpet dust, and floor surface) and their corresponding processing blanks. Processing blanks are defined as sampling media (i.e. sterile filters for air samples) that were processed in a similar manner during the sampling process through PCR amplification. Therefore, processing blanks captures both field and lab contaminants. The hand microbiome of one researcher is also included. (TIF) [file pone.0230700.s009.tif]

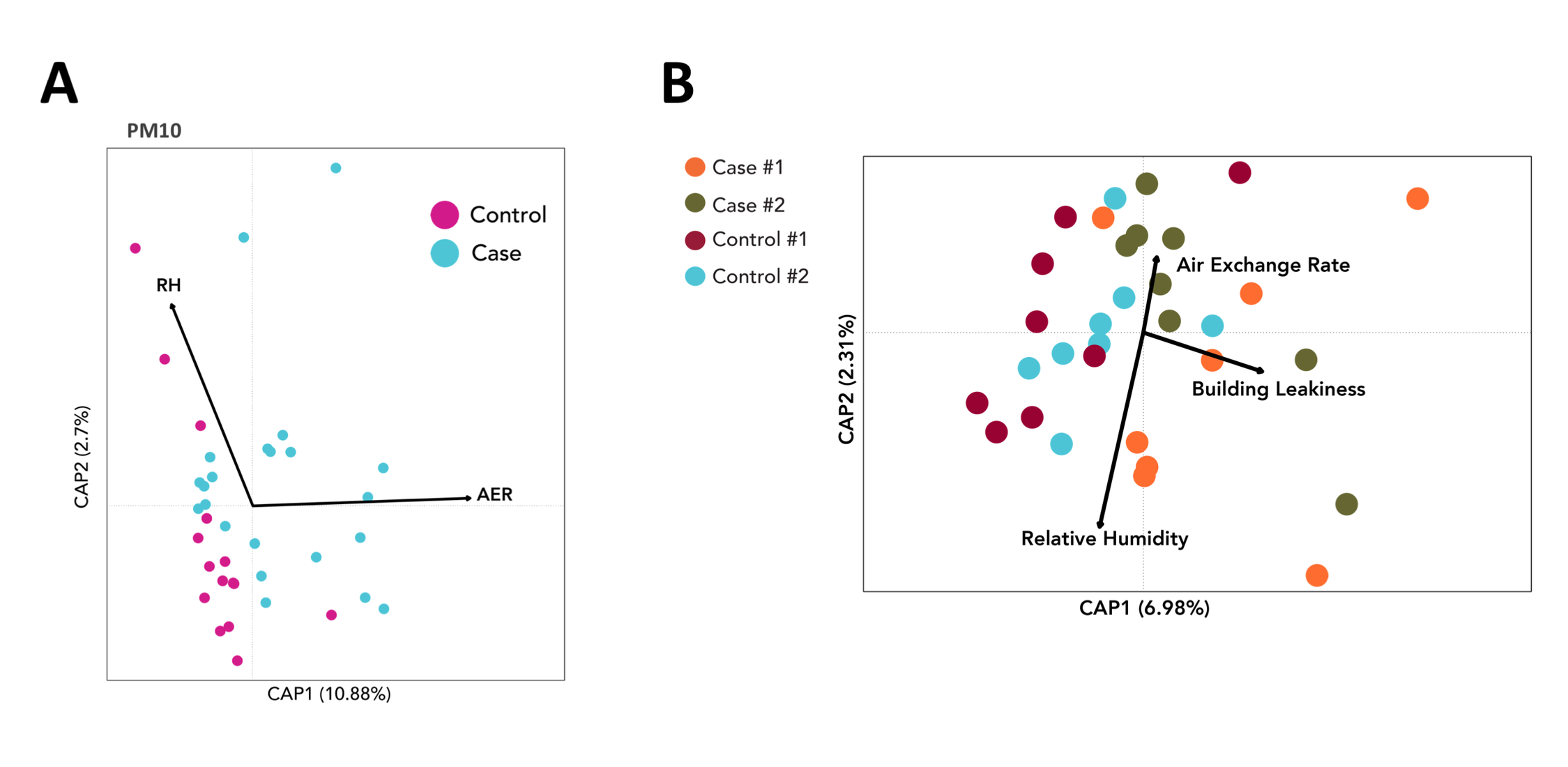

Supplement: S10 Fig — Data from two preliminary analyses of airborne bacterial communities in residential homes. A) A small study of two homes. Each home was sampled multiple times throughout the year, with samples representing every season (Summer, Spring, Autumn, and Winter). Bacterial community was analyzed from PM10, or particulate matter with diameters lower than 10-μm. The results from constraining bacterial community differences against environmental parameters and building characteristics are shown. RH is relative humidity (%); AER is air change rate (h-1). B) Bacterial community analysis of a small group of 4 homes: 2 case and 2 control homes. Air samples were also constrained against environmental parameters and building characteristics. (TIF) [file pone.0230700.s010.tif]

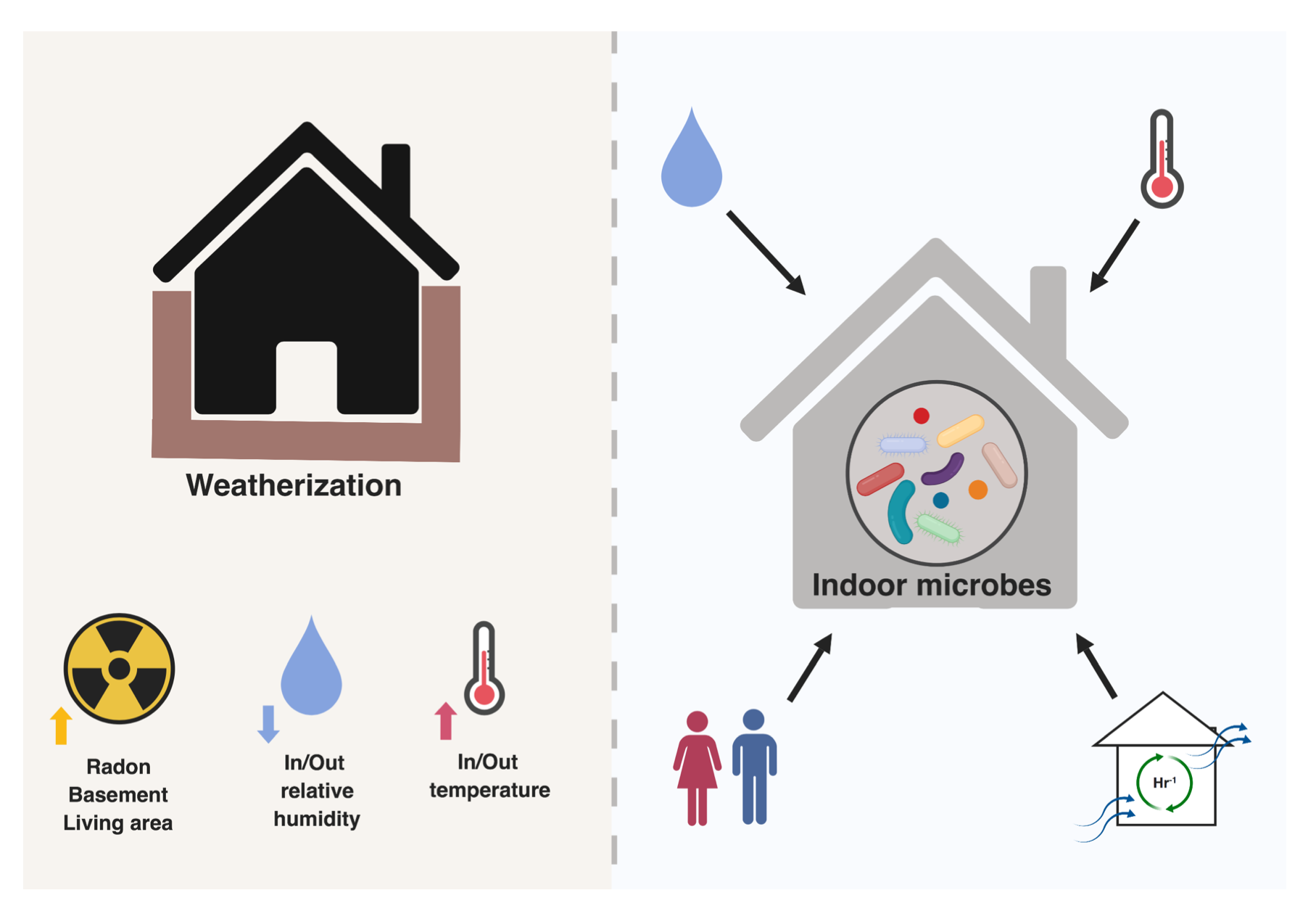

Supplement: S1 Graphical abstract — (TIFF) [file pone.0230700.s015.tiff]
