# Supplementary material for: The short-term effect of residential home energy retrofits on indoor air quality and microbial exposure: A case-control study
Source: PLoS One. 2021 Sep 20;16(9):e0230700. doi: 10.1371/journal.pone.0230700 (PMC8452058; doi:10.1371/journal.pone.0230700)
Supplement: S1 Table — (PDF) [file pone.0230700.s011.pdf]

S1 Table. Sampling timeline of case and control homes.

Sampling timeline (season)<br>1st sampling period – 2nd sampling period	Number of control houses	Number of case houses
Autumn – Spring	3	2
Autumn - Winter	2	1
Winter – Spring	2	2
Summer - Winter	2	2
Summer – Spring	2	2
