# Supplementary material for: The short-term effect of residential home energy retrofits on indoor air quality and microbial exposure: A case-control study
Source: PLoS One. 2021 Sep 20;16(9):e0230700. doi: 10.1371/journal.pone.0230700 (PMC8452058; doi:10.1371/journal.pone.0230700)
Supplement: S2 Table — (PDF) [file pone.0230700.s012.pdf]

S2 Table. Partial Pearson correlation coefficients *r* between ventilation rate, occupancy, and environmental conditions.

	Δ ACH_{CO2}		Δ Occupancy Rate		Δ IO Temperature Gradient	
	Cases	Controls	Cases	Controls	Cases	Controls
Δ Occupancy Rate	-0.38	0.29	--	--	--	--
Δ IO Temperature Gradient	**-0.59***	-0.19	-0.01	0.07	--	--
Δ IO RH Gradient	0.05	-0.01	0.08	0.22	**0.84*****	**-0.79*****

*: $p < 0.05$; **: $p < 0.01$; ***: $p < 0.001$
Δ: defined as value at second sampling event minus value at first sampling event
ACH_{CO2}: natural ventilation *via* CO_2 tracer gas decay
IO Temperature Gradient: ratio of the indoor and outdoor (IO) temperatures, calculated as a time average throughout the sampling period
IO RH Gradient: ratio of the indoor and outdoor (IO) relative humidity, calculated as a time average throughout the sampling period
