# Supplementary material for: The short-term effect of residential home energy retrofits on indoor air quality and microbial exposure: A case-control study
Source: PLoS One. 2021 Sep 20;16(9):e0230700. doi: 10.1371/journal.pone.0230700 (PMC8452058; doi:10.1371/journal.pone.0230700)
Supplement: S1 File — (PDF) [file pone.0230700.s013.pdf]

S1 File: Methods

Study Design

Building and occupant questionnaires were developed from multiple sources, including the Environmental Protection Agency (EPA), Center for Disease Control (CDC), New York State Department of Health, and University of California Berkeley's Center for the Built Environment. We defined weatherization (case) homes as houses that underwent a retrofit. The same company, through the Tompkins Community Action Weatherization Assistance Program, performed all the retrofits for each weatherization home. Retrofit measures for each home included air sealing and a combination of basement and attic insulation. Weatherization homes (n = 9) were sampled before the retrofit, as well as after the retrofit, with at least 3-months between retrofit completion and sampling (**S1 Fig**). In a previous study, 3 weeks has been shown to be an appropriate time to allow for the home environment to stabilize after a retrofit [1]. Here, we extended the waiting period to 3 months to ensure complete stabilization. We defined control homes (n = 11) as houses that did not undergo a retrofit or any structural changes between sampling campaigns. The two sampling campaigns for control homes were also at least 3 months apart. All sampling campaigns for case and control homes were carried out throughout the year, resulting in a balanced sampling timeline between case and control homes (**S1 Table**).

Building leakage

We measured building leakage *via* the blower-door test (Retrotec, Everson, WA) to obtain building leakage measurement in cubic meter *per* h at 50-pascal (CMH_{50}). To ensure no interference with sample collection, blower-door tests were conducted well before each sampling

campaign or after each sampling campaign was completed. Air change rate at 50-pascal (ACH_{50}) was calculated by normalizing CMH_{50} by the building volume as shown in Eq. 1 of the main text

Natural ventilation: During each sampling campaign, the air change rate was measured *via* tracer gas (CO_2) decay to estimate the natural ventilation rates. A 9-kg tank of food-grade CO_2 (Airgas, Elmira, NY) was placed on the first level of the building and programmed to release for two, 15-min intervals *per* day with an automatic gas regulator (Airgas). CO_2 tanks were stabilized for occupant safety on a Radnor® MIG Welding Cart (Airgas). The indoor and outdoor CO_2 level was simultaneously measured with a Li-COR CO_2 Monitor (Li-COR Biosciences, Lincoln, NE) and a SBA-5 CO_2 Gas Analyzer (PP Systems, Amesbury, MA), and recorded with data loggers (HOBO 4-Channel Analog Data Logger, Onset Computer Corporation, Bourne, MA). After sampling, air change rate *per* h was calculated as an average of the decay rates during the 3-4 days sampling period, using the following equation from Laussmann and Helm [2] as sown in Eq. 2 of the main text.

Recording environmental conditions and radon concentration

Both indoor and outdoor environmental conditions were recorded during each sampling period. Indoor temperature and relative humidity in 11 locations inside the house was recorded every min with temperature and relative humidity loggers (HOBO UX100-003 Temperature-Relative Humidity data logger, Onset Computer Corporation). Locations included bedroom areas, bathroom(s), living room area, and the basement. Outdoor temperature and relative humidity were recorded at least one meter away from the house exterior with the HOBO U12-013 data logger (Onset Computer Corporation). Indoor-outdoor ratios for temperature and relative

humidity were calculated by time-matching living room area temperature/relative humidity and outdoor temperature/relative humidity. Lastly, both basement and living area radon levels were measured simultaneously during each sampling campaign. The basement radon levels were measured with the Short Term Liquid Scintillation Kit (AccuStar, Medway, MA) to obtain a time-average. The living room radon levels were measured hourly with the RadStar RS800 Continuous radon monitor (AccuStar). Hourly radon measurements were utilized in the living room to generate informative indoor exposure reports for homeowners.

Microbial sampling and analysis

Dust samples collection

Carpet dust samples were collected using a 3-stage sampling cassette. The 3-stage cassette (Zefon International Inc., Ocala, FL) was assembled in a sterile, DNA-free laminar flow hood with a sterile 37-mm, 0.4-µm-pore size polycarbonate membrane filter (Steriltech Corporation, Kent, WA). During sample collection, the top stage was removed and the outflow was connected to a vacuum pump with a flow rate of 60 lpm. The cassette was held vertically face down on a carpet area while moving horizontally over an approximate 0.01-m^2 carpet area for 30 s. The top stage was then placed back on the cassette for sample storage. Carpet dust samples were taken as close to the center of the carpeted area as possible. If a room had more than one carpet, the carpet that was closer to the room center was selected. If a room had no carpeted area, no dust sample was collected. All air, dust, and floor surface samples were stored at -20°C *prior* to further processing.

Bacterial community analysis

Relationship between samples were visualized using Principal Coordinates Analysis (PCoA) of weighted and unweighted UniFrac distance matrices [3]. To explore the relationship between the indoor microbiome and environmental variables, we performed constrained ordination analysis using the capscale method of the vegan R package [4]. Constrained ordination is a multivariate analysis of community composition and environmental (or explanatory) variables, using ANOVA to determine statistically significant variables that explains differences in community composition. Distance-based redundancy analysis is performed on the weighted UniFrac distance matrix. The capscale function first ordinates the matrix (similar to a PCoA) and then complete a redundancy analysis between the ordination eigenvalues and a set of input constraining (explanatory) variables. Variables that were not significant ($p > 0.05$) or had high collinearity with another variable (variance of inflation factor > 5) were removed from the constraining variables set. The constrained ordination analysis was repeated until all variables in the constraining (explanatory variables) set were significant ($p < 0.05$) and non-collinear (VIF < 5).

S1 File References

1. Pigg S, Cautley D, Francisco P, Hawkins B, Brennan T. Weatherization and indoor air quality: Measured impacts in single-family homes under the Weatherization Assistance Program. Oak Ridge, Tennessee, USA: Oak Ridge National Laboratory, 2014.
2. Laussmann D, Helm D. Air change measurements using tracer gases. In: Mazzeo N, editor. Air change measurements using tracer gases: methods and results Significance of air change for indoor air quality, chemistry, emission control, radioactive pollution and indoor air quality: Books on Demand; 2011.
3. Lozupone C, Knight R. UniFrac: a new phylogenetic method for comparing microbial communities. Applied and Environmental Microbiology. 2005;71(12):8228-35. doi: 10.1128/AEM.71.12.8228-8235.2005. PubMed PMID: PMC1317376; PubMed Central PMCID: PMC16332807
4. Dixon P. VEGAN, a package of R functions for community ecology. Journal of Vegetation Science. 2013;14(6):927. doi: 10.1111/j.1654-1103.2003.tb02228.x.
