# Supplementary material for: The short-term effect of residential home energy retrofits on indoor air quality and microbial exposure: A case-control study
Source: PLoS One. 2021 Sep 20;16(9):e0230700. doi: 10.1371/journal.pone.0230700 (PMC8452058; doi:10.1371/journal.pone.0230700)
Supplement: S2 File — (PDF) [file pone.0230700.s014.pdf]

S2 File: Building/Occupant Survey

PART I. BUILDING INFORMATION

This survey asks several questions regarding your residence that is critical in forming a complete picture of indoor air quality in your home. **All information is treated in the strictest confidence.**

1. Occupancy

Is the basement occupied? Full-time Occasionally Rarely Almost never

What is the general use for each floor?

Level	General use of each floor (family room, bedroom, laundry, workshop, office, storage, etc.)
Basement	______
1st floor	______
2nd floor	______

2. Type of heating system(s) used in this building (circle all that apply— and note primary):

Hot air circulation
Space heaters
Electric baseboard
Wood stove
Hot water baseboard
Other: ________________

3. Primary type of fuel used (circle one):

Natural gas
Electric
Wood
Propane
Coal
Solar

4. Air conditioning (if applicable): Central air Window units Open windows None

5. Is there a kitchen exhaust fan? Yes No

6. Is there a clothes dryer? Yes No

7. Is there an attached garage? Yes No

8. During the past three months, have any of the following taken place in your home?

☐ New carpeting
If so, where and when? ____

☐ Cleaned carpet
If so, where and when? ____

☐ Walls painted
If so, where and when? ____

☐ New furniture (sofa, desks, etc.)
If so, where and when? ____

☐ New wall covering/paper

If so, where and when? ____

☐ Water damage

If so, where and when? ____

☐ Room(s) rearranged

If so, where and when? ____

☐ Pesticide application

If so, where and when? ____

9. Are any of the following regularly used in your home?

	If so, room(s) used in:
☐ Spray disinfectant/deodorizers	____________________
☐ Plug-in or gel air fresheners	____________________
☐ Spray cleaners	____________________
☐ Cosmetic products	____________________
☐ Dry cleaning	____________________
☐ Candles	____________________
☐ Live plants	____________________

10. Are there any persistent odor? If so, when and how often are they notice and can you describe the odor (smells like paint, musty, exhaust, chemicals, etc.)?

____

____

11. Are there any other conditions of note, or other comments which you would like to share? For example, mold growth, pest infection, etc.

____

____

____

____

____

____

____

PART II. OCCUPANT INFORMATION

This survey inquires about each occupant's health and experiences in the home. This information is important in identifying possible correlations between indoor pollutants and health symptoms. **All information is treated in the strictest confidence.**

Name (optional): ____

Age: ______________ Gender (circle one): Female Male

Language fluency (circle all that applies): English Spanish Chinese Other: _________________

Race/ethnicity: Non-Hispanic white Non-Hispanic black Hispanic Asian

1. How many hours do you work/are out of the house per week? ____________
   Do you work on the weekends? Yes No
   1a. Do you use solvents at work? (e.g. chemical manufacturing or laboratory, auto mechanic, painting, cosmetologist) Yes No

2. Which area(s) or room(s) do you spend the most time in the house?

____

____

3. Does any of your daily activities produce dust or odor?

Yes
No

Please explain: ______________________________

____

____

4. Circle the answers that best describe you.

Been diagnosed with migraine?	Yes	No
Have pollen allergies?	Yes	No
Have skin allergies?	Yes	No
Have a cold/flu?	Yes	No
Have sinus problems?	Yes	No
Have asthma?	Yes	No
Do you smoke?	Yes	No
Respiratory disease	Yes	No
Cardiovascular disease	Yes	No

5. Do you believe you are or may be allergic/sensitive to any of the following?

Pollen or plants	Yes	No
Mold	Yes	No
Animal dander (*specify*:______________________)	Yes	No
Dust	Yes	No
Chemicals (*specify*:______________________)	Yes	No
Commercial/personal products (*specify*:______________________)	Yes	No
Other: ______________________	Yes	No

6. Check the symptoms you have experienced while in your home.

**Symptom**	Not at all	Sometimes (a couple of days in the last month)	Occasionally (a couple of days a week in the last month)	Frequently (almost every day)	When you exit the house, these symptoms usually… Got worse	Stayed the same	Got better
Wheezing							
Coughing							
Headache							
Sore, or dry throat							
Chest tightness							
Dry, itching, or irritated eye							
Unusual fatigue, drowsiness, or tiredness							
Dizziness or lightheadedness							
Sneezing							

Nausea or upset stomach							
Dry or itchy skin							
Other: ____________________							

Have you seen a doctor about any of these symptoms? Yes No
If yes, please explain:

7. Do you consider yourself sensitive to the presence of chemicals in the air in your home? Please elaborate.

8. Do you have any concerns about indoor air quality in your home? Please elaborate.
